# Supplementary figures and images for: A Novel SNPs in Alpha-Lactalbumin Gene Effects on Lactation Traits in Chinese Holstein Dairy Cows
Source: Animals (Basel). 2019 Dec 29;10(1):60. doi: 10.3390/ani10010060 (PMC7023285; doi:10.3390/ani10010060)

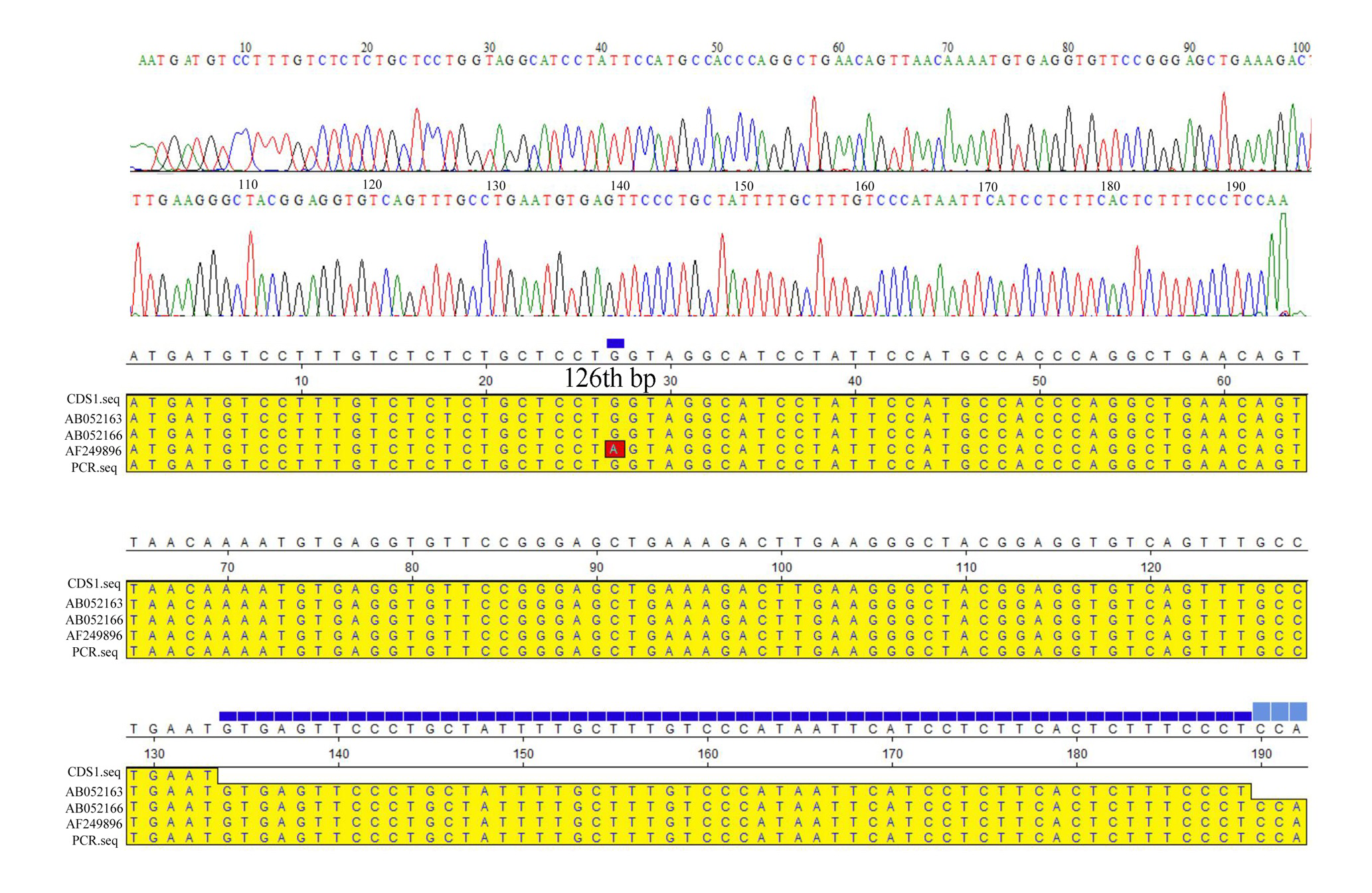

Supplement: Supplementary file 1 [file animals-10-00060-s001.zip › Supplements materials/Figure S1.tif]

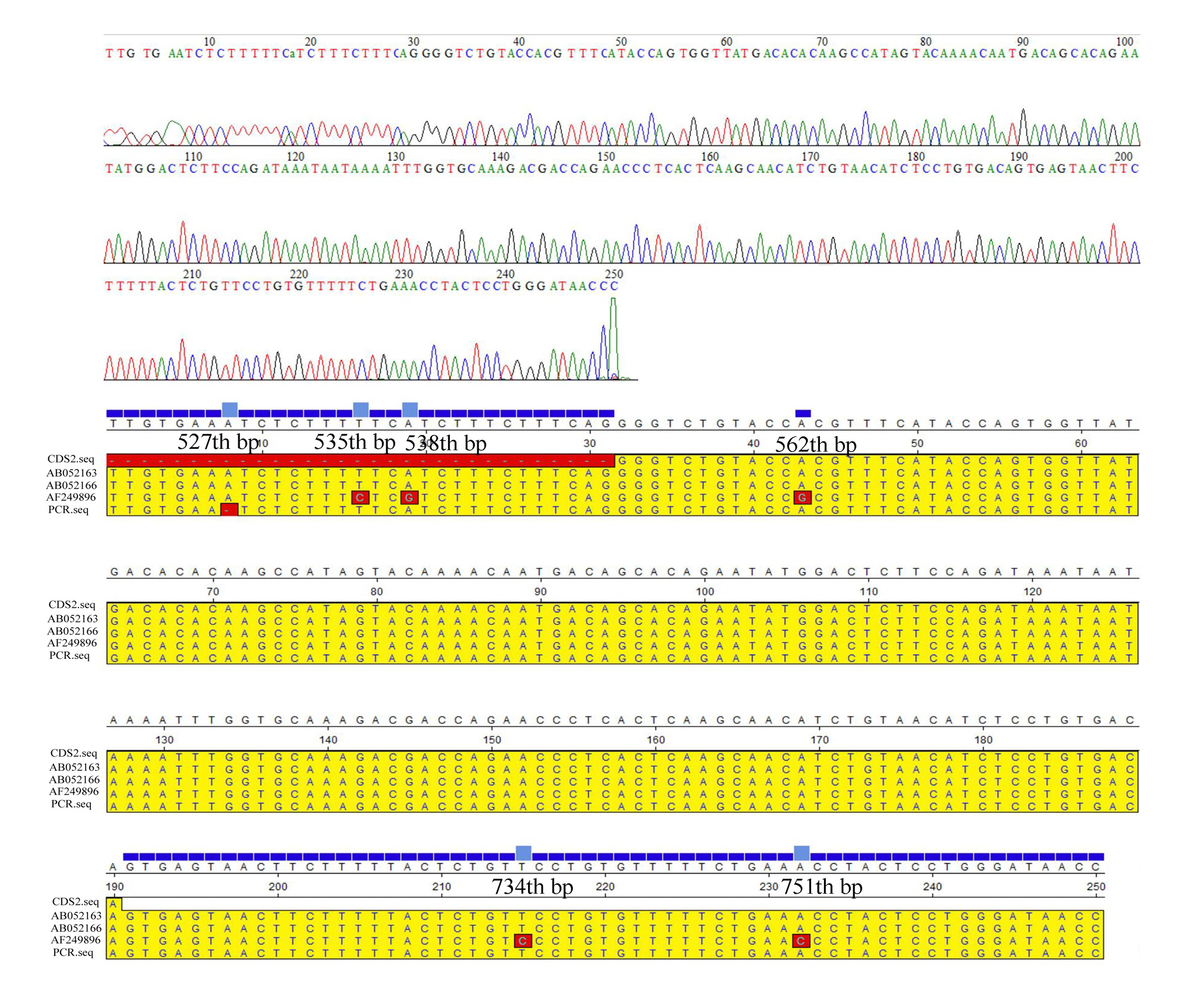

Supplement: Supplementary file 1 [file animals-10-00060-s001.zip › Supplements materials/Figure S2.tif]

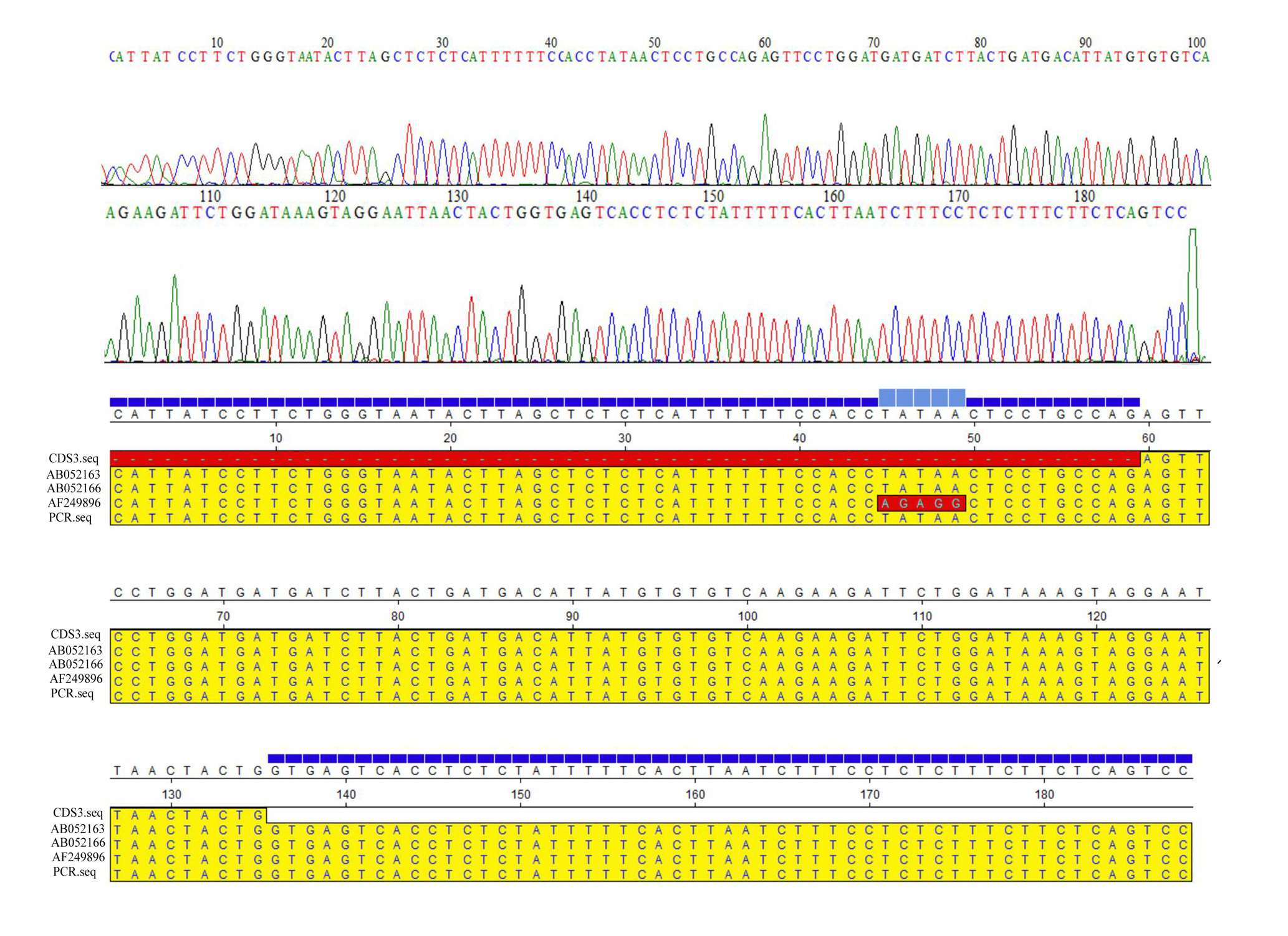

Supplement: Supplementary file 1 [file animals-10-00060-s001.zip › Supplements materials/Figure S3.tif]

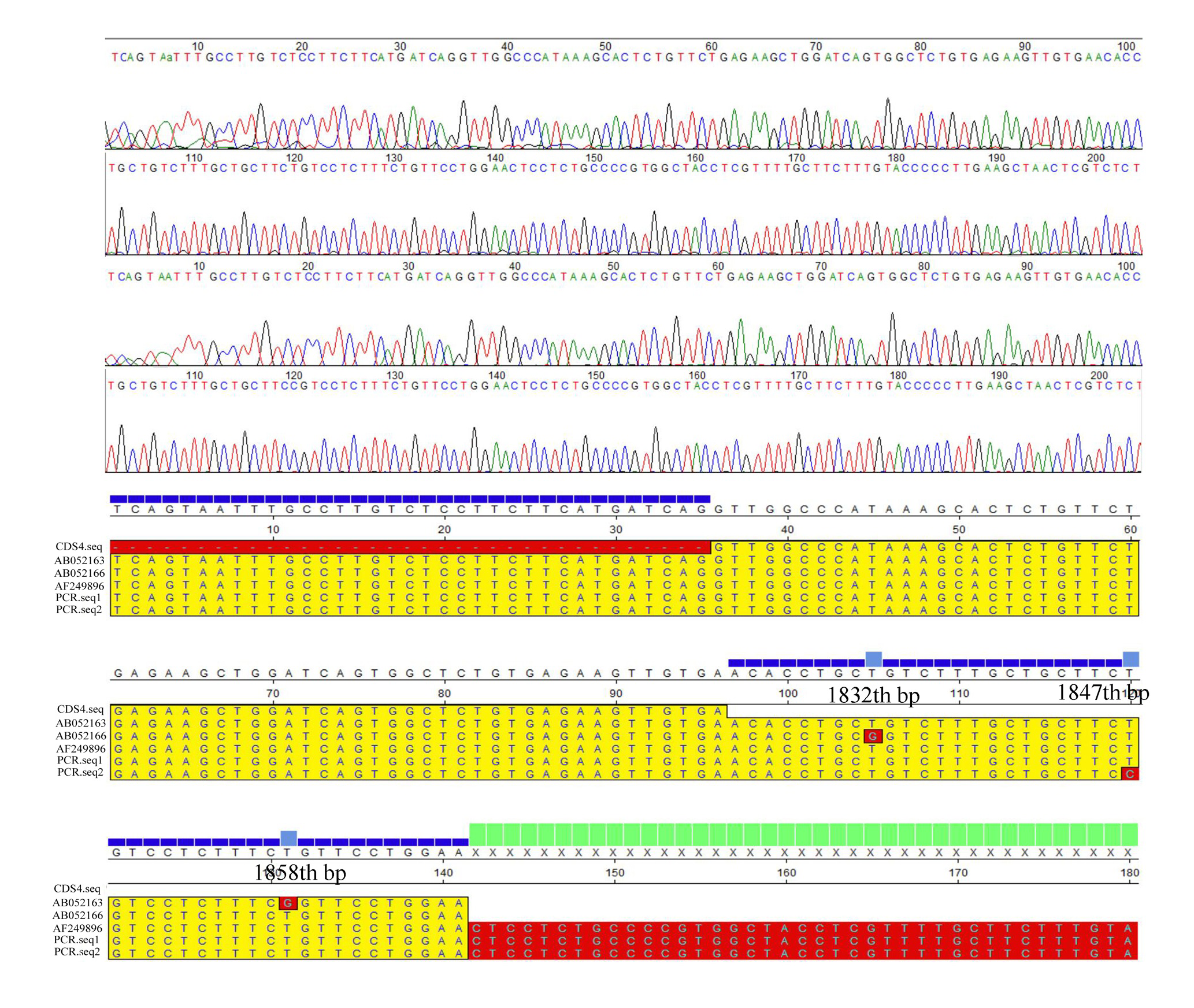

Supplement: Supplementary file 1 [file animals-10-00060-s001.zip › Supplements materials/Figure S4.tif]

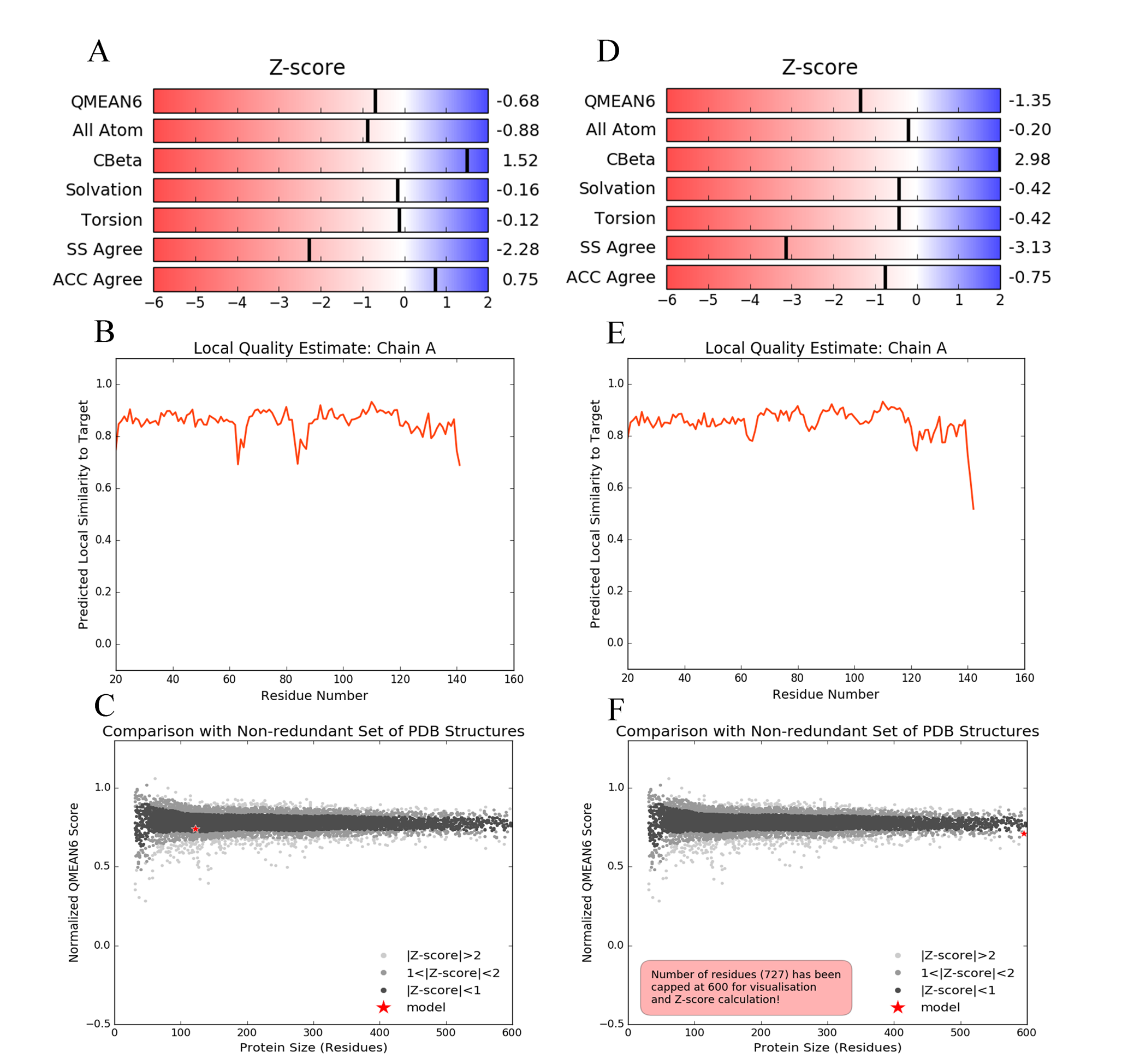

Supplement: Supplementary file 1 [file animals-10-00060-s001.zip › Supplements materials/Figure S5.tif]
